# Crucial role of IL-6, IL-8, IL-11 and leptin in tumor microenvironment in a group of patients with GEP-NETs- crosstalk between inflammation and cancer

**DOI:** 10.3389/fendo.2026.1838186

**Published:** 2026-07-02

**Authors:** Agata Świętek, Joanna K. Strzelczyk, Dorota Hudy, Zenon P. Czuba, Karolina Snopek-Miśta, Mariusz Kryj, Katarzyna Kuśnierz, Marcin Zeman, Malgorzata Oczko-Wojciechowska, Daria Handkiewicz-Junak, Janusz Strzelczyk

**Affiliations:** 1 Department of Medical and Molecular Biology, Faculty of Medical Sciences in Zabrze, Medical University of Silesia in Katowice, Zabrze, Poland; 2 Department of Microbiology and Immunology, Faculty of Medical Sciences in Zabrze, Medical University of Silesia in Katowice, Zabrze, Poland; 3 Department of Oncological Surgery, Prof. Kornel Gibiński Independent Public Central Clinical Hospital, Medical University of Silesia in Katowice, Katowice, Poland; 4 Department of Gastrointestinal Surgery, Faculty of Medical Sciences in Katowice, Medical University of Silesia in Katowice, Katowice, Poland; 5 III Department of Oncological Surgery, Maria Sklodowska-Curie National Research Institute of Oncology, Gliwice Branch, Gliwice, Poland; 6 Department of Clinical and Molecular Genetics, Maria Sklodowska-Curie National Research Institute of Oncology, Gliwice Branch, Gliwice, Poland; 7 Department of Nuclear Medicine and Endocrine Oncology, Maria Sklodowska-Curie Institute National Research Institute of Oncology, Gliwice, Poland; 8 Department of Endocrinology and Neuroendocrine Tumors, Department of Pathophysiology and Endocrinology, Faculty of Medical Sciences in Zabrze, Medical University of Silesia in Katowice, Katowice, Poland

**Keywords:** gastroenteropancreatic neuroendocrine tumors, Interleukin-6, Interleukin-8, Interleukin-11, leptin

## Abstract

**Introduction:**

GEP-NETs constitute a heterogeneous group of rare cancers arising in biologically diverse microenvironments, while their cytokine profiles remain insufficiently characterized.

**Objectives:**

This study assessed IL-6, IL-8, IL-11 and leptin concentrations in tumor and corresponding surgical margins. Also, we aimed to determine the relationship between cytokines concentration and other clinical and demographic variables.

**Patients and methods:**

A multiplex immunoassay and ELISA were applied to quantify cytokine levels in tissue homogenates from 59 patients with GEP-NETs.

**Results:**

The significant differences between tumor and margin samples were identified, with higher concentrations of IL-6, IL-8, IL-11 and leptin in tumor. IL-6 and leptin levels were substantially elevated in tumors and margins from patients with T4. Tumor leptin levels were increased in patients with N2 vs. N0. Margin IL-11 concentration was higher in N0 compared with N2. Tumor IL-6 and leptin level was significantly higher in patients with M1 vs. M0. Moreover, cytokine levels varied according to tumor localization, with differences observed between pancreatic, ileal, colonic and small intestine tumors. Furthermore, IL-6, IL-8, and IL-11 were associated with comorbidities, IL-6 was associated with body mass, while altered leptin patterns appeared to reflect exposure to stimulants such as smoking.

**Conclusions:**

Our study demonstrated altered expression of IL-6, IL-8, IL-11, and leptin in GEP-NET tissues compared with corresponding surgical margins. The cytokines were associated with selected clinicopathological and socio-demographic variables. These findings support the relevance of inflammatory and metabolic signaling within the GEP-NET microenvironment and provide a basis for further studies investigating their potential biomarker value.

## Introduction

Gastroenteropancreatic neuroendocrine tumors (GEP-NETs) represent a heterogeneous group of neoplasms arising from neuroendocrine cells dispersed throughout the gastrointestinal tract and pancreas (1, 2). Although historically considered rare, their incidence has steadily increased over the past decades, likely due to improved diagnostic modalities, heightened clinical awareness, and evolving classification systems (3, 4). GEP-NETs exhibit diverse clinical behaviors ranging from indolent, well-differentiated tumors to highly aggressive, poorly differentiated neuroendocrine carcinomas. Their biological heterogeneity is further reflected in varied secretory profiles, leading to a spectrum of hormonal syndromes and metabolic disturbances (5). Current prognostic stratification relies primarily on tumor differentiation, grading, primary site, and metastatic burden; however, these parameters often fail to fully capture the biological complexity of the disease. As a result, there is a growing interest in identifying additional biomarkers that may better characterize tumor behavior, predict therapeutic response, and refine patient management (6).

Inflammation has emerged as a key modulatory factor in cancer development and progression, with chronic inflammatory signaling increasingly recognized as an enabling hallmark of cancerogenesis (7). Among the diverse inflammatory mediators implicated in tumor biology, cytokines such as IL-6, IL-8, IL-11 and leptin have garnered particular attention due to their involvement in pathways critical to tumor growth and metastatic potential (8). IL-6 is a multifunctional cytokine associated with activation of the JAK/STAT3 pathway, promotion of angiogenesis, and systemic effects such as cachexia and paraneoplastic inflammatory responses (9). Elevated IL-6 levels have been correlated with poor prognosis in several malignancies (10, 11). IL-8, a chemokine primarily known for its role in neutrophil recruitment, also contributes to tumor angiogenesis, epithelial-to-mesenchymal transition, and metastatic dissemination (12). Its expression is often upregulated in tumors characterized by hypoxia, high vascularity, and active stromal remodeling (13). IL-11, another member of the cytokine family, activates overlapping intracellular signaling pathways and is increasingly recognized as a driver of tumorigenesis (14). Leptin, an adipocytokine primarily involved in appetite regulation and metabolic homeostasis, has emerged as a significant mediator linking obesity, chronic inflammation, and cancer (15). Beyond its metabolic functions, leptin can promote cell proliferation, inhibit apoptosis, and enhance angiogenesis through activation of pathways such as JAK/STAT, PI3K/AKT, and MAPK (16).

While the role of inflammatory and metabolic cytokines in oncology is increasingly recognized, their specific profiles in GEP-NETs remain incompletely characterized. Comprehensive analysis of these mediators may yield insights into disease pathophysiology and support the identification of novel prognostic biomarkers. Unlike previous studies primarily utilizing immunohistochemistry or qPCR, this study focuses on the quantification of IL-6, IL-8, IL-11, and leptin protein levels using multiplex immunoassay and ELISA in tumor and matched margin samples. We also examine potential correlations between these proteins and a clinicopathological and socio-demographic variables (TNM status, G status, diabetes, hypertension, BMI, sex, age, smoking status, alcohol consumption status, and biochemical parameters).

## Materials and methods

### Patients and samples

In total, 59 GEP-NET tumor specimens and 43 corresponding surgical margin were collected from patients during surgical resection at the Department of Oncological Surgery, Prof. Kornel Gibiński Independent Public Central Clinical Hospital, Medical University of Silesia in Katowice, Poland; Department of Gastrointestinal Surgery, Faculty of Medical Sciences in Katowice, Medical University of Silesia in Katowice, Poland and the III Department of Oncological Surgery, Maria Sklodowska-Curie National Research Institute of Oncology in Gliwice, Poland. Tumor staging was based on the Version 9 AJC on Cancer Staging System for GEP-NETs (17). The surgical margin samples were checked and classified as cancer-free by pathologists. The main inclusion criteria were: a diagnosis of GEP-NET, written informed consent to participate in the study, age over 18 years, no chronic inflammatory diseases and no history of preoperative radio- or chemotherapy. The serum concentrations data of selected biomarkers (chromogranin A (CgA), serotonin, 5-hydroxyindoleacetic acid (5-HIO) and metabolic parameters (glucose, total cholesterol (TCH), triglycerides (TG) were obtained from hospitals databases (**Supplementary Table 1**). The mean age was 56 ± 12.26, while the average BMI was 27.37 ± 5.30. Details are presented in **Table 1**.

**Table 1:** Clinico-pathological and socio-demographic characteristics of the study group.

Parameter		N (%)
Sex	Female	36 (61.02)
	Male	23 (38.98)
Hypertension	Yes	29 (49.15)
	No	30 (50.85)
Diabetes	Yes	14 (23.72)
	No	45 (76.28)
Smoking	Yes	13 (22.03)
	No	42 (71.19)
	NA*	4 (6.78)
Alcohol drinking	Yes	9 (15.25)
	No	46 (77.97)
	NA*	4 (6.78)
T classification	T1	12 (20.34)
	T2	15 (25.42)
	T3	14 (23.73)
	T4	18 (30.51)
Nodal status (N)	N0	11 (18.64)
	N1	38 (64.41)
	N2	10 (16.95)
Metastasis (M)	0	39 (66.10)
	1	20 (33.90)
Histological grading (G)	G1	42 (71.19)
	G2	15 (25.42)
	G3	1 (1.69)
	NEC	1 (1.69)
Primary tumor location	Ileum	22 (37.29)
	Small intestine	15 (25.41)
	Pancreas	12 (20.34)
	Colon	5 (8.47)
	Other (stomach, duodenum, rectum, appendix, caecum)	5 (8.47)

*NA, not assessed.

**Figure 1:**
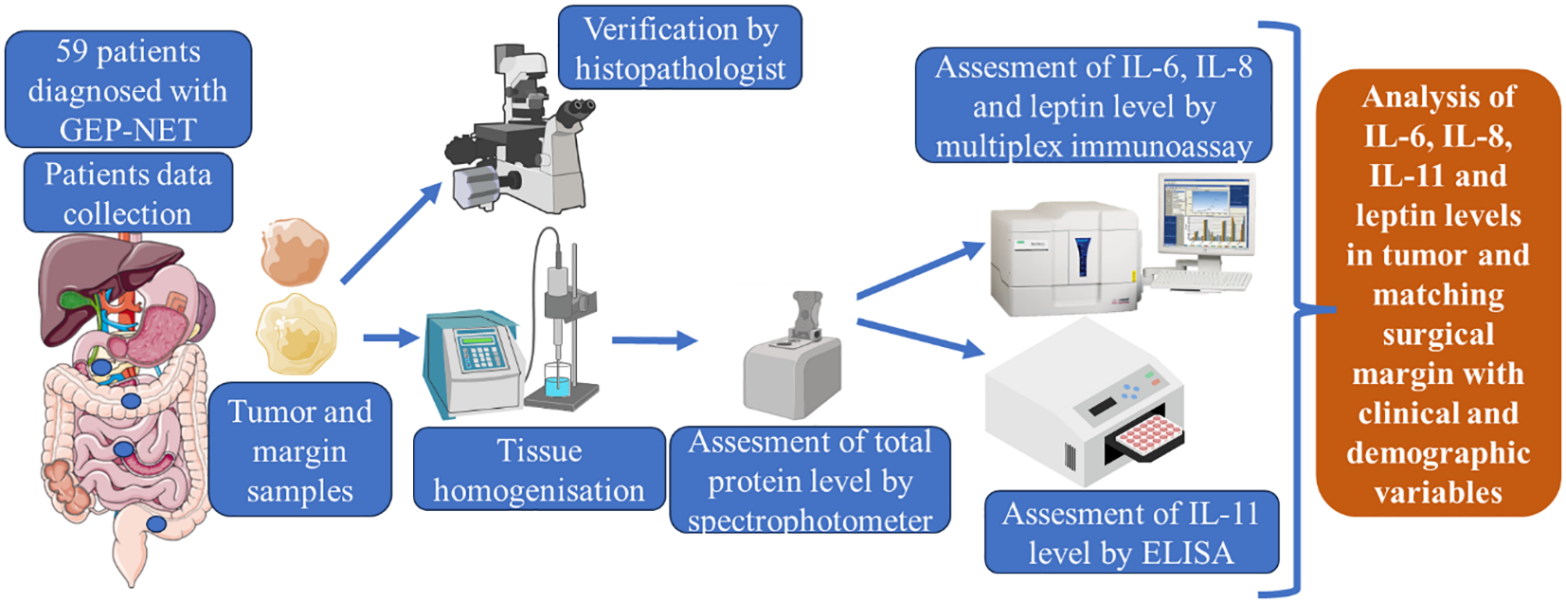
General scheme of the study protocol.

All laboratory analyses were performed at the Department of Medical and Molecular Biology and Department of Microbiology and Immunology, Faculty of Medical Sciences in Zabrze, Medical University of Silesia in Katowice, Poland. The study was approved by the Bioethics Committee of the Medical University of Silesia (approval no. BNW/NWN/0052/KB1/126/I/22/23). The research protocol is presented in **Figure 1**.

### Determination of selected cytokines concentration

The tumor and margin samples were homogenized using a homogenizer Bio-Gen PRO200 (PRO Scientific Inc., Oxford, CT, USA) at a speed of 10,000 RPM for 60 s in nine volumes of cold PBS (EURx, Gdańsk, Poland). Subsequently, the suspensions were sonicated at 100% amplitude, applying three 10-second pulses separated by 20-second intervals on ice between cycles, using the ultrasonic processor UP100H (Hilscher, Teltow, Germany). The homogenized samples were centrifuged at 12,000 rpm for 15 min at 4 °C. The supernatants were collected for further analysis.

Quantification of IL-6, IL-8, and leptin was performed using the Human Magnetic Luminex Assay Multiplex Kit (R&D Systems, Inc., Minneapolis, MN, USA) according to the manufacturer’s protocols. The assay is based on fluorescence-encoded magnetic microspheres coated with analyte-specific monoclonal antibodies, providing high sensitivity and specificity with low sample volume requirements. Assay plates, standards, controls, and reagents were prepared as instructed, and standard dilutions covering the full dynamic range were generated using kit-provided reference standards, with blank wells included for background determination. Magnetic beads for IL-6, IL-8, and leptin were incubated with samples, standards, and controls, followed by sequential addition of detection antibodies and streptavidin-phycoerythrin. Signal acquisition was performed on a Bio-Plex 3D Suspension Array System using Luminex xMAP Technology with Luminex xPONENT version 4.3.309.1 for FLEXMAP 3D (Luminex Corporation, Austin, TX, USA). Cytokine concentrations were calculated using Bio-Plex Manager Software version 6.2 (R&D Systems, Inc., Minneapolis, MN, USA) with five-parameter logistic (5-PL) standard curve fitting.

Enzyme-linked immunosorbent assay (ELISA) was used to evaluate IL-11 protein concentrations in tissue homogenates according to the manufacturer’s instructions (Assay ID: SEA057Hu, Cloud-Clone Corp., Houston, TX, USA). The 450 nm wavelength was used to record the absorbance, and this was calibrated according to the standard curve using Synergy H1 microplate reader (BioTek, Winooski, VT, USA) and results were calculated with Gen5 2.06 software (BioTek, Winooski, VT, USA). The sensitivity of this assay was 6.0 pg/mL. The coefficient of variation was measured to be less than 10% (intra-assay) and less than 12% (inter-assay) by precision measurement.

Total protein concentration was determined using a NanoDrop ND-1000 UV/VIS spectrophotometer (NanoDrop Technologies, USA) by measuring absorbance at 280 nm with automatic path-length correction, using the corresponding homogenization buffer as blank. In addition, total protein was quantified in 15 randomly selected samples with a fluorescence-based assay (Accu Orange Protein Quantitation Kit, Biotium, Fremont, CA, USA). Fluorescence was measured at 480 nm excitation and 598 nm emission using a SYNERGY H1 microplate reader (BIOTEK, Winooski, VT, USA) operated with Gen5 2.06 software. The two methods showed no significant differences. Cytokine concentrations were normalized to total protein content and expressed as pg/mg protein. All measurements were performed in duplicate.

### Statistical analyses

Data distribution was evaluated using the Shapiro–Wilk test. Depending on the distribution and assumptions, differences between two groups were analyzed using the Mann–Whitney U test or Student’s t-test to compare medians or means, respectively. Differences between tumor and margin were tested with Wilcoxon signed-rank test. For comparisons involving more than two groups, the Kruskal–Wallis test followed by Bonferroni *post hoc* analysis was applied. Associations between variables were examined using Spearman’s correlation coefficient. Multiple linear regression was done to determine if more than one parameter can affect the results. Age, BMI, alcohol, smoking status, diabetes, hypertension, TNM status, G status and location were assessed simultaneously. Fisher exact test was used to determine associations of demographic data with location of the tumor. Statistical significance was defined as p < 0.05. Results are reported as median with interquartile range (M [Q1–Q3] 95%CI) or as mean ± standard deviation (M ± SD 95%CI), reported p-values are from Bonferroni *post hoc* test or Mann-Whitney U/Student’s t-test. Patients with missing data were excluded from analysis that considered the missing parameter. Significant results are presented with an asterisk on figures. Summary of used tests and made comparisons are presented in **Supplementary Table 2**.

## Results

Our analysis showed statistical differences in the expression levels of IL-6, IL-8, IL-11 and leptin proteins in the tumor as compared to the surgical margin in a group of patients with GEP-NET, (**Table 2**).

**Table 2:** Concentration of the selected cytokines in tumor samples and margin samples in a group of patients with GEP-NET.

Analyzed cytokines	Protein concentration [pg/mg] [median (lowest - highest quartile)]		p
	Tumor	Margin	
IL-6	2.69 [1.84-4.65] (95%CI 2.16-3.85)	1.49 [1.18-2.17] (95%CI 1.30-2.04)	<0.001
IL-8	10.77 [6.37-21.06] (95%CI 9.19-19.35)	5.96 [3.35-7.54] (95%CI 4.29-6.54)	<0.001
IL-11	6.44 [4.97-8.47] (95%CI 4.75-10.79)	3.25 [1.27-6.68] (95%CI 0.84-7.16)	0.0342
Leptin	52.80 [35.82-84.92] (95%CI 42.21-69.99)	25.28 [17.78-44.82] (95%CI 20.12-40.62)	<0.001

**Figure 2:**
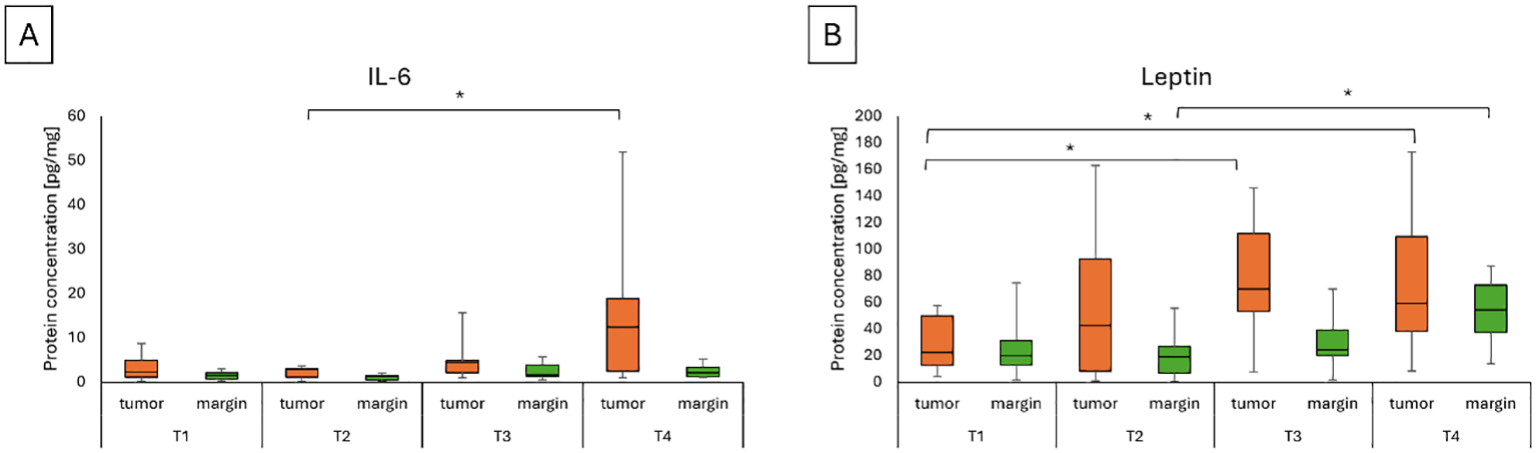
Results of the proteins level analyses in tumor and margin samples compared to T status. **(A)**—The IL-6 level in the tumor and margin samples in the group of patients with T1 (12 patients), T2 (15 patients), T3 (14 patients) and T4 (18 patients) status; **(B)** — The leptin level in the tumor and margin samples in the group of patients with T1 (12 patients), T2 (15 patients), T3 (14 patients) and T4 (18 patients) status. * p-value<0.05.

The level of IL-6 in tumors was significantly higher obtained from patients with T4 compared to T2 status (12.37 [2.43-18.87] (95%CI 2.71-15.47) vs. 2.69 [1.08-3.08] (95%CI 0.60-3.24); p=0.01), (**Figure 2A**).

Higher leptin concentration was noted in tumors, in group with T1 compared to samples with other T parameters; T1 vs. T4 (22.14 [12.53-49.83] (95%CI 5.56-53.00) vs. 59.05 [38.09-109.53] (95%CI 39.33-107.52); p=0.049) and T1 vs. T3 (22.14 [12.53-49.83] (95%CI 5.56-53.00) vs. 70.22 [53.36-111.64] (95%CI 53.36-111.64); p=0.022). Higher leptin level was observed in margins, in group with T4 status compared to T2; T4 vs. T2 (54.22 [37.70-72.78] (95%CI 35.67-74.28) vs. 18.99 [6.57-26.56] (95%CI 3.60-45.20); p=0.029), (**Figure 2B**).

**Figure 3:**
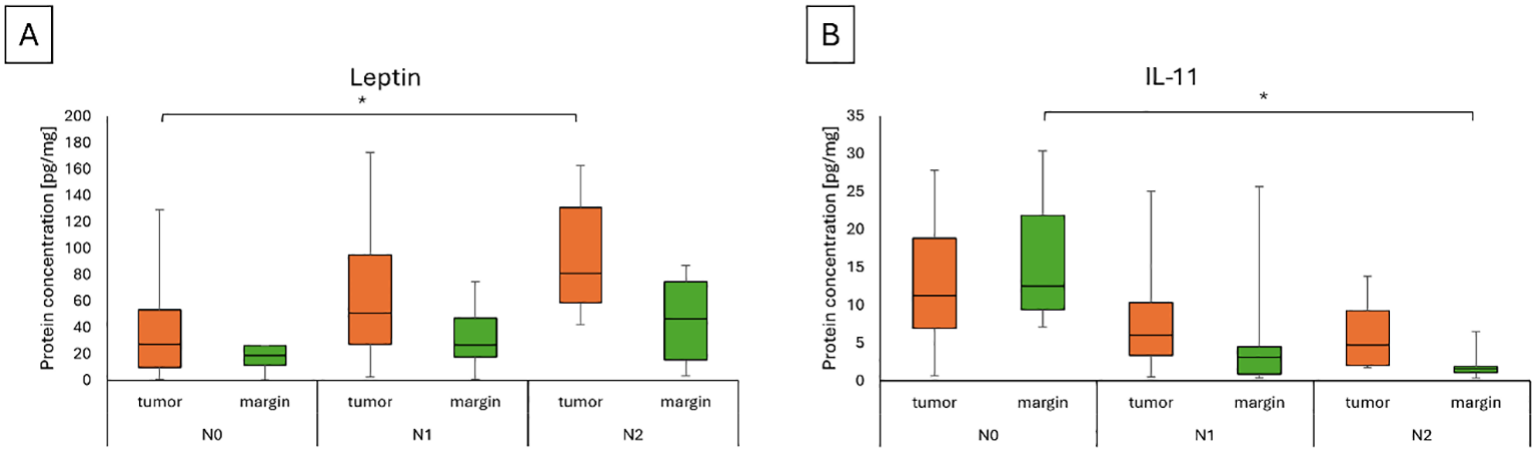
Results of the proteins level analyses in tumor and margin samples compared to nodal status. **(A)**—The leptin level in the tumor and margin samples in the group of patients with N0 (11 patients), N1 (38 patients), and N2 (10 patients) status; **(B)** — The IL-11 level in the tumor and margin samples in the group of patients with N0 (11 patients), N1 (38 patients), and N2 (10 patients) status. * p-value<0.05.

Higher leptin level was reported in the tumors, collected from patients with N2 in comparison to the N0 status (81.30 [59.05-130.89] (95%CI 55.67-145.68) vs. 27.42 [9.97-53.36] (95%CI 5.56-81.10); p=0.013), (**Figure 3A**). Higher IL-11 concentration was observed in margins, in a group with N0 compared to N2 (N0 vs. N2: 12.57 [9.45-21.88] (95%CI 7.16-30.36) vs 1.60 [1.12-1.86] (95%CI 0.39-6.52); p=0.019), (**Figure 3B**).

**Figure 4:**
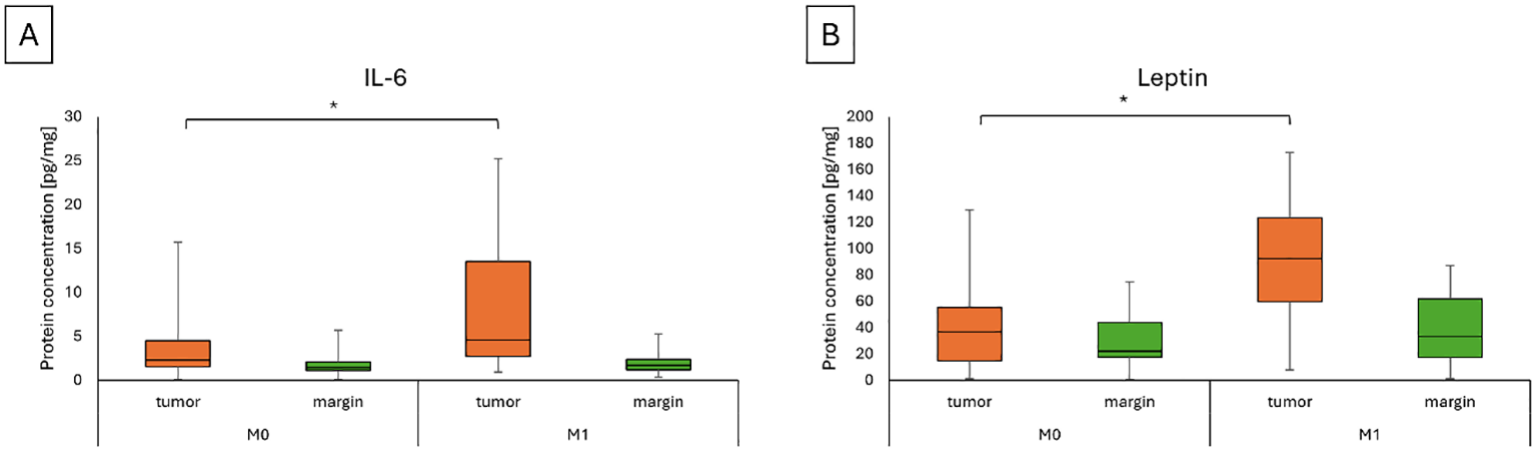
Results of the proteins level analyses in tumor and margin samples compared to metastasis status. **(A)**—The IL-6 level in the tumor and margin samples in the group of patients with M0 (39 patients) and M1 (20 patients) status; **(B)** — The leptin level in the tumor and margin samples in the group of patients with M0 (39 patients) and M1 (20 patients) status. * p-value<0.05.

In tumor samples, significantly higher IL-6 level was observed in patients with M1 compared to stage M0 (4.65 [2.70-13.53] (95%CI 2.71-13.43) vs. 2.28 [1.60-4.53] (95%CI 1.77-3.68); p=0.007), (**Figure 4A**).

Higher concentration of leptin was observed in tumors, in patients with M1 in comparison to M0 status (92.13 [59.82-123.30] (95%CI 62.12-115.17) vs. 36.75 [15.14-55.31] (95%CI 25.90-52.04); p<0.001, (**Figure 4B**).

**Figure 5:**
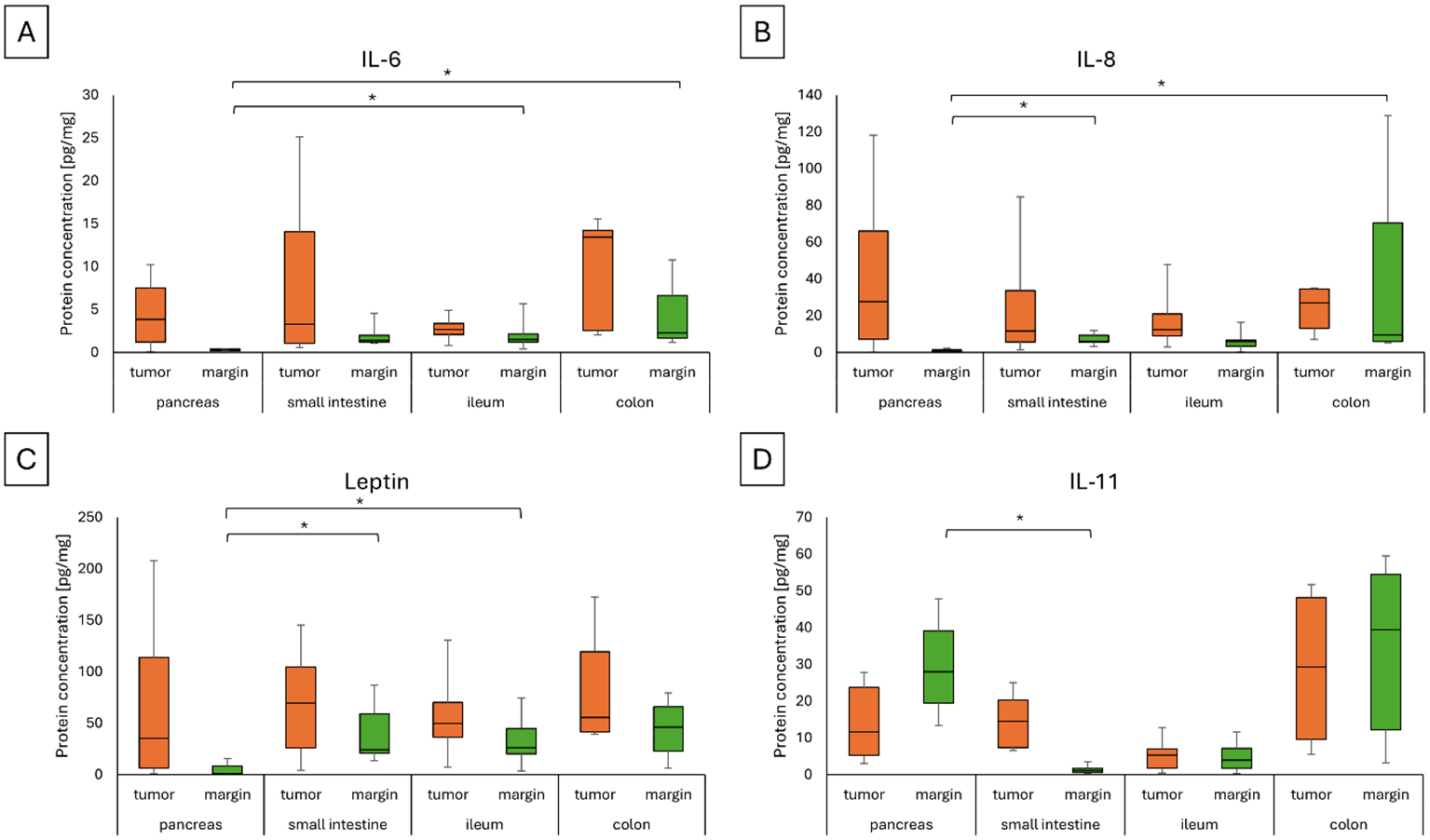
Results of the proteins level analyses in tumor and margin samples compared to localization of the primary tumor. **(A)**—The IL-6 level in the tumor and margin samples in the group of patients with tumor located in pancreas (12 cases), small intestine (15 cases), ileum (22 cases) and colon (5 cases); **(B)**— The IL-8 level in the tumor and margin samples in the group of patients with tumor located in pancreas (12 cases), small intestine (15 cases), ileum (22 cases) and colon (5 cases); **(C)**— The leptin level in the tumor and margin samples in the group of patients with tumor located in pancreas (12 cases), small intestine (15 cases), ileum (22 cases) and colon (5 cases); **(D)**— The IL-11 level in the tumor and margin samples in the group of patients with tumor located in pancreas (12 cases), small intestine (15 cases), ileum (22 cases) and colon (5 cases). * p-value<0.05.

Higher IL-6 concentration was found in margins, obtained from patients with primary tumors located in the ileum or colon, compared to pancreas (ileum vs. pancreas: 1.54 [1.23-2.16] (95%CI 1.32-2.12) vs. 0.29 [0.20-0.40] (95%CI 0.15-0.46); p=0.017), (colon vs. pancreas: 2.29 [1.68-6.62] (95%CI 1.19-10.82) vs. 0.29 [0.20-0.40] (95%CI 0.15-0.46); p=0.010), (**Figure 5A**).

Also higher IL-8 level was noted in margins, obtained from patients with primary tumors located in the colon compared to pancreas (9.65 [6.05-70.67] (95%CI 5.33-128.81) vs. 0.82 [0.26-1.76] (95%CI 0.08-2.34); p=0.011). A similar relationship was observed in the case of locations in the small intestine and pancreas (6.26 [5.75-9.30] (95%CI 5.55-10.85) vs. 0.82 [0.26-1.76] (95%CI 0.08-2.34); p=0.026), (**Figure 5B**).

In margins, the concentration of leptin was higher in patients whose primary tumors were located in the ileum or small intestine compared with the pancreas (ileum vs. pancreas: 26.56 [20.22-45.07] (95%CI 20.49-44.70) vs. 1.43 [0.83-8.83] (95%CI 0.46-16.00); p=0.030), (small intestine vs. pancreas: 24.24 [21.09-59.28] (95%CI 17.68-71.29) vs. 1.43 [0.83-8.83] (95%CI 0.46-16.00); p=0.042), (**Figure 5C**).

Higher concentration of IL-11 was observed in margins, in patients with primary tumor located in pancreas compared with the group with location in small intestine (pancreas vs. small intestine: 28.00 [19.52-39.10] (95%CI 13.40-47.84) vs. 1.12 [0.59-1.79] (95%CI 0.39-3.59); p=0.007, (**Figure 5D**).

**Figure 6:**
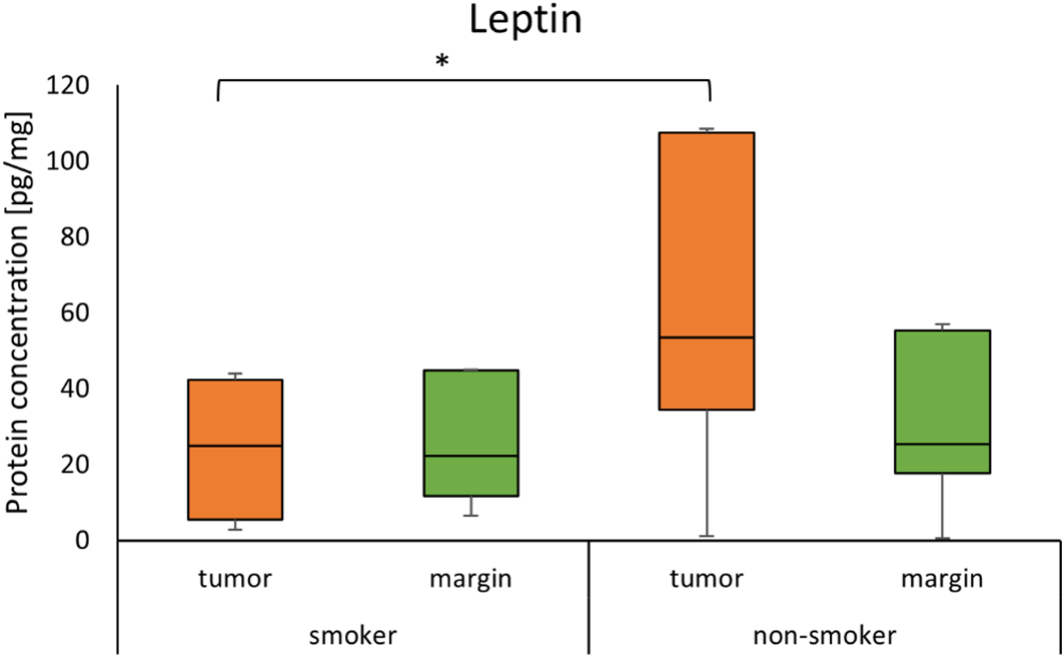
Results of the leptin level analyses in tumor and margin samples compared to smoking status (13 vs. 42 patients). * p-value<0.05.

The median concentration of leptin was higher in tumors, in the non-smoker individuals as compared with smokers (53.36 [34.36-107.52] (95%CI 45.93-81-10) vs. 24.88 [5.56-42.21] (95%CI 5.56-42-21); p=0.004), (**Figure 6**).

Higher IL-6 levels in tumor, in the non-diabetic group compared to those with diabetes (4.68 [2.82-18.87] (95%CI 2.93-15.47) vs. 2.49 [1.73-3.93] (95%CI 1.72-4.18); p=0.023). In margins, in the hypertensive group, IL-6 level was elevated, compared to the non-hypertensive group (2.01 [1.32-3.89] (95%CI 1.32-3.89) vs. 1.19 [0.28-1.32] (95%CI 0.15-2.17); p=0.017). In margins, in the hypertensive group, IL-8 level was elevated compared to those without hypertension (6.78 [4.89-11.14] (95%CI 5.33-10.85) vs. 1.87 [0.33-5.53] (95%CI 0.08-6.05); p=0.006). In tumors, IL-11 was elevated in the non-diabetic and non-hypertensive group compared to those with both conditions (11.66 [6.55-13.25] (95%CI 3.11-14.59) vs. 1.98 [1.70-4.75] (95%CI 0.61-6.02); p=0.026).

In tumor, an increased level of IL-6 was noted in group of patients with normal weight compared to overweight (4.71 [2.70-15.55] (95%CI 2.71-15.47) vs. 2.16 [0.81-3.32] (95%CI 0.70-4.18); p=0.025).

The analysis of cytokine levels revealed multiple significant associations. In the tumors, IL-6 correlated with IL-8 (r_S_ = 0.76, p<0.05) and leptin (r_S_ = 0.50, p<0.05). IL-8 correlated with leptin (r_S_ = 0.53, p<0.05). Leptin correlated with serotonin (r_S_=0.402, p=0.027). IL-11 correlated with 5-HIO (r_S_=-0.66, p=0.019). In turn, in the margins, IL-6 correlated with IL-8 (r_S_ = 0.73, p<0.05) and leptin (r_S_ = 0.78, p<0.05). IL-8 correlated with leptin (r_S_ = 0.60, p<0.05), 5-HIO (r_S_= 0.645, p=0.013), glucose (r_S_=0.0398, p=0.02) and TG (r_S_=-0.53, p=0.025). Moreover, cross-compartment correlations showed that IL-6 in tumors correlated with margins IL-8 (r_S_ = 0.37, p=0.03). Margins IL-6 correlated inversely with tumors IL-11 (r_S_ = –0.47, p=0.041). Margins IL-8 correlated inversely with tumors IL-11 (r_S_ = –0.48, p<0.005).

To determine if some parameters have been affecting concentration of studied cytokines we performer multiple linear regression analysis. For margin tissues two of analyzed cytokines were significantly affected by age (IL-11) and tumor G status (IL-8) while in tumor tissue IL-6 was most correlated with M and G status, IL-8 with G status, IL-11 was determined by location of the tumor and leptin was dependent of M and smoking status. Detailed results are presented in the **Table 3**.

**Table 3:** Results of multiple linear regression analysis.

Analyzed cytokines	Tissue	Best fitted predictors	R^2^	R^2^ adjusted	b_0_	F	p
IL-6	tumor	M status (b=18.04; p=0.030); G status (b=13.09; p=0.044)	0.16	0.127	-9.00; p=0.357	4.78	0.013
	margin	G status (b=-3.26; p=0.515)	0.01	-0.02	11.11; p=0.136	0.43	0.515
IL-8	tumor	G status (b=130.49; p<0.001)	0.22	0.2	-110.64; p=0.035	14.13	<0.001
	margin	G status (b=12.44; p=0.018)	0.14	0.12	-3.09; p=0.679	6.1	0.018
IL-11	tumor	Location* (b=-4.24; p=0.048)	0.15	0.11	23.96; p<0.001	4.33	0.048
	margin	Age (b=-0.45; p=0.018)	0.24	0.2	34.36; p=0.003	6.61	0.018
Leptin	tumor	M status (b=57.03; p<0.001); Smoking status (b=-39.36; p=0.021)	0.3	0.28	56.00; p<0.001	10.91	<0.001
	margin	Smoking status (b=150.65; p=0.054)	0.1	0.07	34.07; p=0.361	3.96	0.054

*Location was presented as 0-small intestine; 1-pancrease; 2-colon; 3-ileum; 4-other.

We observed no other statistically significant differences between the concentration of analyzed cytokines and clinicopathological parameters or sociodemographic parameters.

## Discussion

Previous immunohistochemical studies of pancreatic NETs (pan-NETs) and gastro-intestinal NETs (GI-NETs) observed higher IL-6 immunoexpression in the peritumoral area, which correlated with disease aggressiveness (18). A similar relationship was obtained in other study, which confirmed high immunoexpression in GEP-NET tumors of gastric, duodenal, ileal, appendicitis and colonic origin (19). Other immunohistochemical studies, in groups of patients with gastritis, gastric adenoma, and gastric cancer, noted expression of IL-6 and a fibroblast marker in stromal cells in all groups (20). A study on pancreatic cancer cell lines suggested that IL-6 stimulates VEGF expression and promotes angiogenesis and tumor growth (21). A meta-analysis of numerous studies on colorectal cancer showed that elevated IL-6 levels influence key processes such as activation of the JAK/STAT3 pathway, promotion of EMT, migration, and invasion of cancer cells (22). In serum-based studies of GEP-NET and BP-NET patients, authors observed significantly elevated circulating levels of IL-6 compared to healthy group (23, 24). In our cohort, IL-6 concentrations were significantly higher in tumor tissue than in matched surgical margins. This finding is consistent with previous observations in GEP-NETs and other gastrointestinal malignancies, supporting the association between local inflammatory activity and tumor presence. However, the present study does not allow determination of the cellular source of IL-6 or its direct functional effects within the tumor microenvironment.

In studies of pan-NETs, ​​immunohistochemistry demonstrated positive IL-8 protein expression in 21% of the analyzed tumors. Furthermore, the authors examined IL-8 gene expression by qRT-PCR and observed almost 44-fold higher expression in tumor tissues compared to healthy tissue, concluding that IL-8 is involved in modulating tumor behavior (25). In a serum study, Geisler et al. demonstrated elevated IL-8 levels in a group of NET patients compared to controls (24). Due to the lack of data, the discussion also included the results of studies on other types of gastrointestinal cancers. Similar results were obtained in qRT-PCR analysis of IL-8 expression in colorectal cancer tissues, which confirmed elevated IL-8 expression in all tumors compared to margin tissues (26). Moreover, meta-analysis confirmed that IL-8 is typically overexpressed in colorectal tumors and can be secreted by both tumor cells and stromal elements, depending on the genetic background of the tumor (27). In another study, the authors noted increased IL-8 immunoexpression in over 50% of analyzed pancreatic adenocarcinoma samples compared to control tissues, therefore concluding a paracrine role of IL-8 in tumor progression and angiogenesis (28). Chen et al. showed that in pancreatic ductal adenocarcinoma, the action of the IL-8/CXCR1 complex influenced the properties of cancer cells, whose behavior was similar to the cancer stem cell population (CSC), defined as a subset of cells within the tumor that initiate and sustain tumor formation and growth due to their self-renewal and differentiation capabilities (28). In our study, the higher IL-8 levels observed in tumor tissue are consistent with previous reports describing increased IL-8 expression in gastrointestinal malignancies. Although functional mechanisms were not investigated in the present study, these findings support a potential association between IL-8 expression and the tumor microenvironment.

Previous studies demonstrated that IL-11 is a key driver of gastrointestinal tumorigenesis and may play an even more prominent role than IL-6 in sustaining STAT3 activation in tumor (29). In mouse models of gastric cancer, excessive IL-11 signaling was shown to promote epithelial cell proliferation and tumor growth, whereas genetic or pharmacological inhibition of IL-11 signaling significantly reduced tumor development (30). Further studies in gastrointestinal cancers confirmed that IL-11 supports tumor cell survival, angiogenesis, and stromal remodeling, particularly through activation of cancer-associated fibroblasts and sustained STAT3 phosphorylation within the tumor microenvironment (31, 32). Based on Pubmed and Medline, we found no studies analyzing IL-11 in GEP-NET. Elevated IL-11 concentrations in tumor tissue compared with margins may reflect differences in local inflammatory and stromal activity. Given the lack of previous studies evaluating IL-11 in GEP-NET tissues, this observation should be interpreted cautiously and requires independent validation.

In colon cancer, immunohistochemistry demonstrated moderate to strong leptin expression in over 90% of the analyzed tumors (33). Data from *in vitro* and *in vivo* experiments have shown that leptin promotes pancreatic cancer cell invasion by inducing metalloproteinases and is involved in tumor progression (34). In turn, another pancreatic cancer study using human and murine cell lines demonstrated that leptin contributes to tumor growth by activating the PI3K/AKT pathway, which promotes tumor cell migration (35). A meta-analysis examining the role of adipokines, including leptin, in gastrointestinal cancers summarized evidence that leptin from peritumoral adipose tissue may influence EMT, angiogenesis, and immune cell recruitment, in tumors located close to adipose tissue, such as those in the intestine or pancreas (36). Although there are no other studies directly examining leptin in GEP-NET tissues, numerous studies on colorectal and pancreatic cancers confirm that leptin can be locally overexpressed. In several studies, mean leptin level in serum and plasma was statistically lower in NET samples, compared to controls (37–40).This phenomenon in our study may result from paracrine activation by adipocytes and autocrine action of tumor cells, promoting proliferation, angiogenesis, and increased invasiveness. However, the present data do not allow conclusions regarding the underlying mechanisms responsible for this association.

In other study, the authors demonstrated that IL-6 immunoexpression increases with tumor grade in GEP-NETs (19). Another study on pancreatic ductal adenocarcinoma cell lines reported that pancreatic stellate cells can secrete IL-6 *via* the STAT3 pathway in tumor cells, which supports their invasiveness (41). Another article on pancreatic cancer studies in a mouse model demonstrated that IL-6 activates the MAPK pathway and supports tumor progression, which may suggest that elevated IL-6 levels promote tumor growth and aggressiveness. A meta-analysis confirmed that IL-6 can support progression in GEP-NETs (42). Similar findings were reported in serum-based NET studies, where elevated circulating IL-6 concentrations were associated with advanced tumor stage, higher tumor burden and more aggressive disease phenotype (24). The association between higher IL-6 levels and advanced disease stage observed in our cohort is consistent with previous reports linking inflammatory cytokines with tumor progression, by activating proinflammatory and proproliferative signaling pathways. Nevertheless, causal relationships cannot be inferred from this cross-sectional analysis.

In an immunohistochemical study in colon cancer, leptin expression was reported to correlate with clinical features such as tumor size, invasion, and metastasis (33). A tissue microarray study in colorectal cancer showed that high leptin immunoexpression in tumor tissue correlated with poorer clinical outcomes, including metastasis grade and lymph node involvement (43). Similar results were obtained in study, where leptin immunoexpression in colorectal cancer significantly correlated with tumor grade 2. Furthermore, leptin immunoexpression in tissues adjacent to the tumor was similar to the level observed in the tumor (44). In a study of the pancreatic cancer cell line PANC1, leptin overexpression was observed, which positively correlated with features such as tumor growth and lymph node metastasis (34). Trevellin et al., in a study analyzing esophageal adenocarcinoma, reported that peritumoral adipose tissue can secrete leptin, which acts in a paracrine manner on tumor cells and promotes invasion and lymph node involvement (45). Previous serum analyses demonstrated that higher leptin concentrations in NET patients were associated with unfavorable clinicopathological features and may reflect enhanced tumor progression and metastatic potential (40). Consistently, elevated leptin levels have previously been linked with metastatic NET disease, systemic inflammation and poorer clinical status of patients (37). Taken together, our observations are consistent with data from other articles describing different types of cancer, where leptin in tumor promotes tumor cell proliferation and migration. The association between elevated leptin levels and adverse clinicopathological features observed in this study is in line with findings reported in several analyses focusing on different types of gastrointestinal cancers. Further studies are needed to clarify whether leptin has prognostic relevance in GEP-NETs.

In our study, we demonstrated differences between the concentration of the analyzed cytokine and the location of the primary tumor. Higher levels of IL-6 and IL-8 in the margins of tumors located in the ileum and colon compared to the pancreas may result from the physiologically stronger immunological and proinflammatory activity of the intestinal wall, which is rich in immune cells, cytokines, and receptors recognizing pathogens (46, 47). The intestinal microenvironment promotes faster activation of proinflammatory pathways, which in the context of the tumor may enhance local production of IL-6 and IL-8 by stromal cells and fibroblasts (26, 27, 46). In the pancreas, however, the microenvironment is less immunologically active, which may explain the significantly lower levels of these cytokines (48). A similar mechanism may explain the results observed with leptin. Intestinal tissues are characterized by a greater number of stromal cells capable of secreting leptin (49). In the pancreas, its poor adipocyte population may promote lower leptin production in the margins (50). In contrast, an inverse relationship has been demonstrated for IL-11, which is closely associated with fibrosis and fibroblast activation; therefore, its levels may be higher in the margins of tissues with a higher stromal and fibrogenic content, such as the pancreas (51). The small intestine, which is less prone to fibrosis, may naturally exhibit lower local IL-11 production, even in the setting of a tumor response (52). The observed differences according to tumor location highlight the biological heterogeneity of GEP-NETs and suggest that local tissue context may contribute to cytokine expression patterns.

Nagayasu et al. showed that smoking-related adipose tissue inflammation leads to downregulation of leptin expression, indicating a direct inhibitory effect of tobacco exposure on leptin regulation (53). These findings are supported by a systematic review and meta-analysis, which confirmed significantly lower serum leptin levels in smokers compared with non-smokers across multiple populations (54). Other data indicate that smoking cessation is associated with an increase in circulating leptin concentrations, further supporting the suppressive effect of active smoking on leptin levels (55). Overall, the higher leptin concentrations observed in non-smokers compared to smokers may reflect the absence of smoking-related suppression of leptin expression and secretion. These findings are consistent with previous observations linking smoking with reduced leptin levels and indicate that smoking status should be considered when interpreting leptin measurements.

Differences in cytokine levels between patients may be due to the impact of chronic diseases such as diabetes and hypertension on systemic and local inflammation. These conditions modify immune activity and the tumor microenvironment, which may lead to different IL-6, IL-8, and IL-11 secretion profiles in tumor and margins (19).

Furthermore, higher IL-6 levels in normal-weight compared with overweight individuals may be related to differences in the metabolic and inflammatory activity of adipose tissue (7). Excess body weight promotes chronic, low-grade inflammation, which potentially influences the regulation of local cytokine responses within the tumor (36).

The observed correlations among IL-6, IL-8, IL-11, and leptin levels highlight the functional interdependence of inflammatory and metabolic pathways within the GEP-NET tumor microenvironment, might suggesting their coordinated involvement in tumor maintenance and stromal remodeling (25, 29, 34). Additionally, the associations between leptin and serotonin, IL-11 and 5-HIO, as well as IL-8 with 5-HIO, glucose, and triglycerides, may indicate a close interaction between cytokine activity, systemic metabolic status, and neuroendocrine signaling. Together, these findings support the concept that cytokine expression in GEP-NETs is influenced not only by local tumor-related factors but also by broader metabolic and neuroendocrine mechanisms (36, 47, 54). However, these associations should not be interpreted as evidence of direct biological interactions.

## Conclusion

This study demonstrated significantly higher concentrations of IL-6, IL-8, IL-11, and leptin in GEP-NET tumor tissues compared with corresponding surgical margins. The cytokines were associated with tumor stage, nodal involvement, metastatic status, primary tumor location, smoking status, and selected comorbidities. These findings support the presence of distinct inflammatory and metabolic profiles within the GEP-NET microenvironment. However, due to the cross-sectional design and limited sample size, the biological and clinical significance of these associations remains to be established. Larger prospective studies are required to determine whether these cytokines have diagnostic, prognostic, or predictive value in group of patients with GEP-NETs.

## Data Availability

The raw data supporting the conclusions of this article will be made available by the authors, without undue reservation.

## Supplementary material

Supplementary Table 1 Biochemical characteristics of the study group. Me, median; Q1, lower quartile; Q3, upper quartile.

Supplementary Table 2 Summary of used test and comparisons.
