# Supplementary material for: Crucial role of IL-6, IL-8, IL-11 and leptin in tumor microenvironment in a group of patients with GEP-NETs- crosstalk between inflammation and cancer
Source: Front Endocrinol (Lausanne). 2026 Jul 2;17:1838186. doi: 10.3389/fendo.2026.1838186 (PMC13372631; doi:10.3389/fendo.2026.1838186)
Supplement: Supplementary Table 1 — Biochemical characteristics of the study group. Me, median; Q1, lower quartile; Q3, upper quartile. [file Table1.docx]

**Table S1.** Biochemical characteristics of the study group.

| **Parameters** | **Study Group Me (Q1–Q3)** |
| --- | --- |
| **Chromogranin A [µg/L]** | 75.37 (6.42-139.75) |
| **Serotonin [ng/mL]** | 500.90 (169.96- 1000.00) |
| **5-hydroxyindole acetic acid (5-HIAA) [mg/24 h]** | 6.50 (4.46-13.60) |
| **Glucose [mg/dL]** | 102.35 (90.70-113.56) |
| **Total cholesterol (TCH) [mg/dL]** | 187.00 (161.70-217.75) |
| **Triglycerides (TG) [mg/dL]** | 104.00 (83.90-139.00) |

Me—median; Q1—lower quartile; Q3—upper quartile

**Table S2.** Summary of used test and comparisons.

| **Comparison parameter** | **Groups** | **Test** | **Correction for multitesting** |
| --- | --- | --- | --- |
| Tissue | tumor vs. margin | paired Wilcoxon | No |
| Sex | male vs. female | U Mann Whitney | No |
| T | T1 vs. T2 vs. T3 vs. T4 | Kruskall Wallis | Bonferroni *post hoc* |
| N | N0 vs. N1 vs. N2 | Kruskall Wallis | Bonferroni *post hoc* |
| M | M0 vs. M1 | U Mann Whitney | No |
| G | G1 vs. G2; G3 and NEC excluded - one case only for each group | U Mann Whitney | No |
| Smoking status | smoking vs. non-smoking | U Mann Whitney | No |
| Alcohol status | abstinent vs. drinking | U Mann Whitney | No |
| Location | pancrease vs. small intestine vs. ileum vs. colon | Kruskall Wallis | Bonferroni *post hoc* |
| Diabetes | diabetes true vs. diabetes false | U Mann Whitney | No |
| Hypertension | hypertension true vs. hypertension false | U Mann Whitney | No |
| Diabetes and hypertension | none vs. both vs. hypertension only; diabetes only excluded - one case only | Kruskall Wallis | Bonferroni *post hoc* |
| BMI | underweight vs. normal vs. overweight vs. obesity | Kruskall Wallis | Bonferroni *post hoc* |
